# Derivation and characterization of human embryonic stem cells on human amnion epithelial cells

**DOI:** 10.1038/srep10014

**Published:** 2015-05-07

**Authors:** Dongmei Lai, Yongwei Wang, Jian Sun, Yifei Chen, Ting Li, Yi Wu, Lihe Guo, Chunsheng Wei

**Affiliations:** 1 The International Peace Maternity and Child Health Hospital, School of medicine, Shanghai Jiaotong University, Shanghai, China; 2 Eye and ENT Hospital, Fudan University, Shanghai, China

## Abstract

Culture conditions that support the growth of undifferentiated human embryonic stem cells (hESCs) have already been established using primary human amnion epithelial cells (hAECs) as an alternative to traditional mitotically inactivated mouse embryonic fibroblasts (MEFs). In the present work, inner cell masses (ICM) were isolated from frozen embryos obtained as donations from couples undergoing *in vitro* fertilization (IVF) treatment and four new hESC lines were derived using hAECs as feeder cells. This feeder system was able to support continuous growth of what were, according to their domed shape and markers, undifferentiated naïve-like hESCs. Their pluripotent potential were also demonstrated by embryoid bodies developing to the expected three germ layers *in vitro* and the productions of teratoma *in vivo*. The cell lines retained their karyotypic integrity for over 35 passages. Transmission electron microscopy (TEM) indicated that these newly derived hESCs consisted mostly of undifferentiated cells with large nuclei and scanty cytoplasm. The new hESCs cultured on hAECs showed distinct undifferentiated characteristics in comparison to hESCs of the same passage maintained on MEFs. This type of optimized culture system may provide a useful platform for establishing clinical-grade hESCs and assessing the undifferentiated potential of hESCs.

Human embryonic stem cells (hESCs) are derived from the inner cell mass (ICM) of embryonic blastocysts. They are a virtually unlimited source of cells that can be stably propagated in an undifferentiated pluripotent state. They hold considerable promise for tissue engineering and drug discovery applications. Ever since the first human embryonic stem cell line was derived, substantial progress has been made in the understanding of stem cell biology, pluripotency, and control of differentiation 1,2,3,4.

However, major technical obstacles still interfere with attempts to characterize and stabilize hESCs in conventional culture systems 2. Most hESC lines were produced using mouse embryonic fibroblasts (MEFs) as feeder cells. This involves considerable risk of contamination and cross-species pathogen transfer. These issues limit the safety and both the research and clinical usefulness of affected hESCs 3. In addition, recent studies have shown that culture conditions may also affect the characteristics of hESCs 4,5.

The derivation of hESC lines has trailed developments in the study of mouse embryonic stem cells (mESCs), which were first established as cell lines in 1981 6,7. Although hESCs and mESCs are both derived from blastocyst-stage embryos, they have very different biological properties with respect to underlying mechanisms of self-renewal 8. Molecular analysis has shown that hESCs retain their pluripotency to a degree and in a manner similar to that of mouse-derived epiblast stem cells (EpiSCs) in most existing culture systems. Hanna *et al.* reported that exogenous factors can cause the pluripotent state to stabilize, allowing the lines to retain the molecular and functional characteristics of naïve pluripotent cells 4.

In our previous work, human amnion epithelial cells (hAECs) served as feeder cells and successfully maintained the undifferentiated growth of mESCs and hESCs. The gene expression, epigenetic modification, and morphology of hESCs cultured with hAECs differed from those cultured with mouse embryonic fibroblasts (MEFs) 9,10,11. Our groups have further shown that hAECs can maintain the pluripotency and undifferentiated growth of EpiSCs. It was found to be possible to reprogram mESCs maintained on hAECs to adopt naïve-like pluripotent traits 12. These findings suggest that suitable conditions are important to maintain the pluripotency of hESCs.

In order to use hESCs for therapeutic applications, such as regenerative medicine, it is important to develop quality humanized culture environments that support derivation, expansion, and differentiation. hAECs have many advantages over mitotically inactivated MEFs. They are isolated from human placental amina, which are usually discarded as medical waste. hAECs also grow slowly and do not express telomerase 13. For this reason, neither mitomycin C nor gamma irradiation treatments are necessary. This makes hAECs ideal feeder cells capable of supporting the undifferentiated growth of embryonic stem cells (ESCs) without competing with them for nutrients.

In the present study, the derivation and propagation of hESC lines using hAECs as a feeder layer are described. Characterization of these hESC lines, including visualization of their fine structures was also performed.

## Results

### Derivation and characterization of hESC lines on hAECs

**Figure 1:**
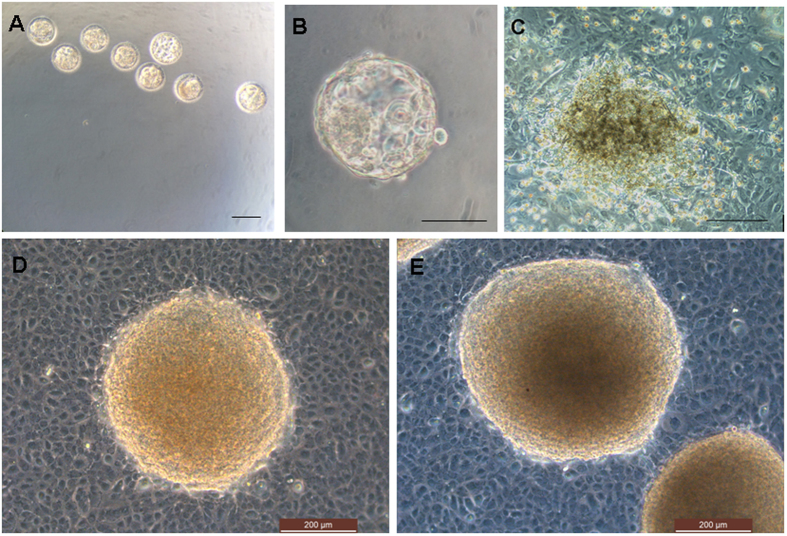
Human embryonic stem cells (hESCs) derivation from pronase-treated and hatched blastocysts. (**A**,**B**) Blastocysts before pronase treatment. (**C**) Outgrowth of pronase-treated blastocyst in hAECs, 7 days after pronase treatment. (**D**) Colony of hESCs derived from the ICM in (**B**). (**E**) Dome-like hESC colony after passage 6. Bar: A, B = 100 μm; C, D, E = 200 μm.

hESC lines are usually derived via immunosurgery to isolate the ICM from the human blastocyst 14,15. Here, hESC lines from protease-treated and hatched blastocysts were established as described previously 16. One to two weeks after plating, 8 expanded ICMs were transferred to fresh, hAEC-coated hESC culture dishes. ES-like outgrowth cells were visible after successful propagation of the ICM, but differentiated cells either died or disappeared. Ultimately, 4 hESC lines were established successfully and the efficiency of the derivation process is 50% (Fig. 1).

**Figure 2:**
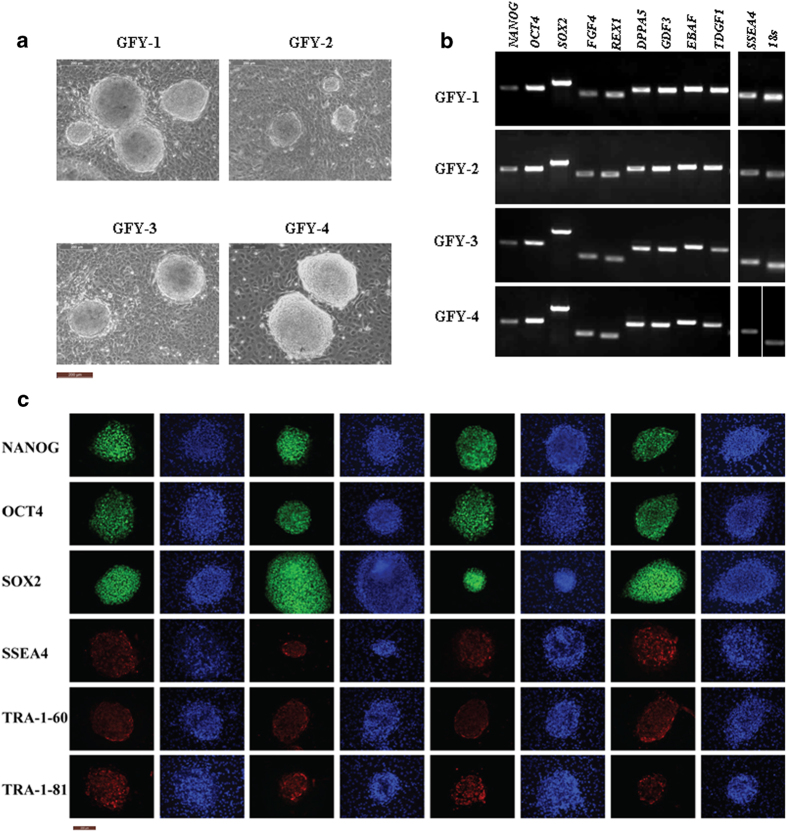
Four hESC lines were derived and established on hAEC feeder layers. (**A**) Morphology of hESC clones were observed via microscopy. Distinguishable borders could be clearly observed. The hAECs were separated from human placentas and used directly as feeder cells directly. (**B**) Stem cell marker genes were detected by RT-PCR. 18s RNA was used as the internal standard control. (**C**) Immunostaining was performed to detect undiferentiated genes. The hESCs were cultured on hAECs and treated with specific antibodies against NANOG, OCT4, SOX2, SSEA4, TRA-1-60, and TRA-1-81. They then were stained with FITC-conjugated secondary antibodies. Nuclei were counterstained with 4’,6-diamidino-2-phenylindole (DAPI). Bars: A, C = 200 μm.

Four hESC lines were established using hAECs as feeder cells. These are here called GFY-1, GFY-2, GFY-3, and GFY-4. As in previous studies, hESCs grown on hAECs aggregated into compact, dome-like colonies with clean, smooth edges 10,11. These colonies were different from monolayer colonies maintained on MEFs (Fig. 2A). In order to analyze the pluripotency and immortality of these cells, the expression of certain marker genes specific to ESCs was detected using RT-PCR (Fig. 2B). Transcripts of those marker genes, which include *NANOG*, *OCT4*, *SOX2*, *FGF4, REX1*, *DPPA5*, *GDF3*, *ERAF*, *TDGF1*, and *SSEA4*, were clearly detected in each cell line. Next, immunostaining was used to examine the expression of undifferentiated marker proteins, such as NANOG, OCT4, SOX2, SSEA4, TRA-1-60, and TRA-1-81 (Fig. 2C). Results showed that all cell lines expressed stem cell marker proteins, indicating that their undifferentiated growth could be supported by hAEC cells.

The population doubling time and single-cell cloning efficiency of the newly derived human ESCs maintained on hAECs or MEFs have been assayed. The results showed that there is no difference of the population doubling time between human ESC on hAECs and that on MEFs, however, single-cell cloning efficiency of the newly derived ESCs maintained on hAECs is higher than that on MEFs ( Fig. S1).

**Figure 3:**
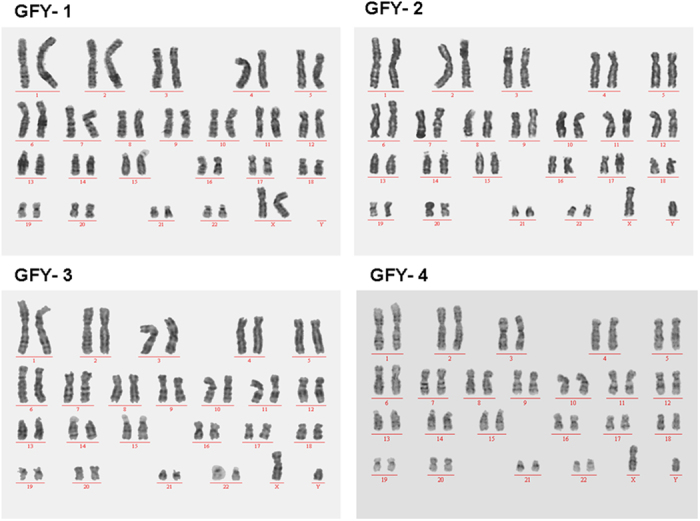
Karyotypes of hESCs were analyzed by the G-band method.

Karyotype analysis demonstrated that chromosomal stability could be maintained well after sequential propagation for 35 passages. As shown in Fig. 3, results indicated normal, stable karyotypes in the four cell lines. These were 46 and XX in GFY-1 and the others were 46 and XY. Thus far, these cell lines have been propagated for about 40 passages, and the morphology of the colonies has not changed. After freezing and thawing, the pluripotency-related genes expression of these cell lines did not change significantly (data not shown).

### Analysis of pluripotency of hESC lines on hAECs

**Figure 4:**
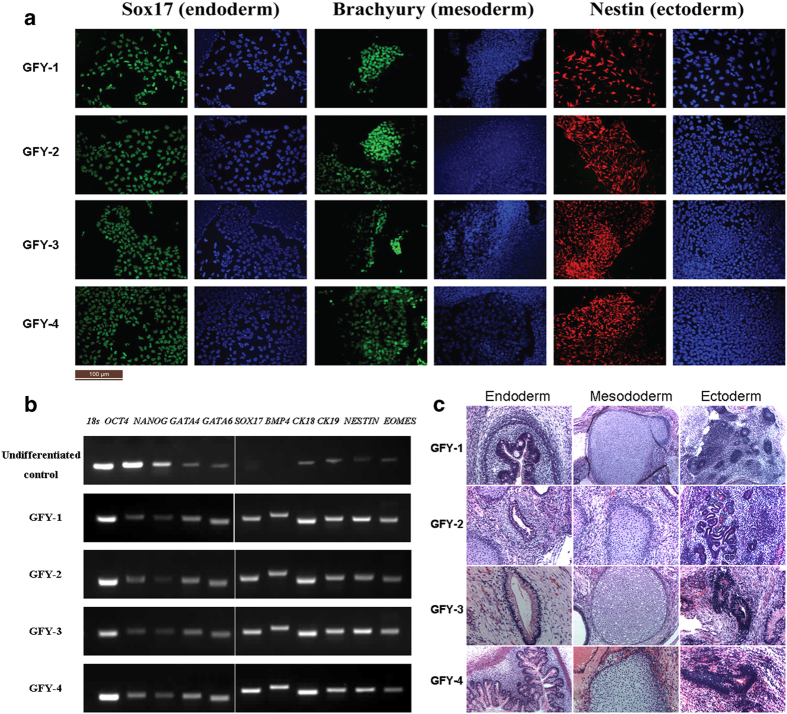
The newly derived hESCs could differentiate into lineages of the three germ layers, both *in vitro* and *in vivo*. (**A**) Immunofluorescent staining detected expression of Sox17 (endoderm), Brachyury (mesoderm), and Nestin (ectoderm). Bar = 100 μm. (**B**) Marker genes associated with the three germ layers were detected by RT-PCR. Amplified DNA segments were separated in agarose gels and visualized by ethidium bromide and 18s RNA was used as the internal standard control. (**C**) Teratomas formed after injection of our four hESC lines into SCID mice. These teratomas contained tissue components of all three embryonic germ cell layers, including the endoderm (intestinal mucosa), mesoderm (cartilage), and ectoderm (neuro epithelium). Original magnification × 100.

The potential to differentiate into the three germ layers is another important feature of ESCs. The developmental capabilities of the four cell lines were determined both *in vitro* and *in vivo*. The hESC clones were picked and allowed to differentiate in suspension in differentiating medium and form embryoid bodies. The embryoid bodies were then transferred to gelatin-treated plates and grown for another 8 days. Differentiated cells, which grew out of the embryoid bodies, were highly heterogeneous in morphology. Fig. 4A showed that these cells were specifically stained with antibodies against Sox17 (endoderm), Brachyury (mesoderm), and Nestin (ectoderm), indicating that differentiated cells from these hESCs expressed representative markers of the three germ layers. RT-PCR showed marker genes associated with all three germ layers in cells expanded from developed embryonic bodies, including: *GATA4*, *SOX17, CK18* and *CK19* (endoderm); *GATA6* and *BMP4* (mesoderm); *NESTIN* (ectoderm) and *NOMES* (trophectoderm). *OCT-4* and *NANOG* are transcription factors essential to the establishment of ESCs from the ICM 1,6,7. As in previous reports 1, undifferentiated hESCs were found to express *OCT-4* and *NANOG*, but these two genes were downregulated concomitant with differentiation (Fig. 4B).

To evaluate the pluripotent potential of these four established hESC lines grown on hAECs, hESC clones were injected into the hind legs of SCID mice. All of the four hESC lines grown on hAECs produced typical immature teratomas containing all three main germ layers. These teratomas contained immature glands (endoderm), cartilage (mesoderm), and neural tubes (ectoderm) (Fig. 4C), as identified by histological analysis.

These results demonstrated that these four hESC lines are capable of differentiating into progeny representing the three embryonic germ layers, both *in vitro* and *in vivo*.

### Ultrastructure analysis of hESC on hAECs

As shown above and reported previously 10,11, when maintained on hAECs, the morphology of hESC colonies was different from that of hESC colonies maintained on MEFs. When grown with hAECs, hESC colonies appeared dome-like and compact and grew from the center toward the periphery. However, it has been reported that undifferentiated hESCs form single-cell layered colonies in culture. Their morphology becomes irregular as the colonies grow 17. Transmission electron microscopy (TEM) was used to analyze the fine structure of the hESC colonies derived and maintained on hAECs.

The ultrastructures of human and murine ESCs maintained on MEFs have been visualized and several differences between undifferentiated mouse and human ESCs can be observed via electron microscopy examination 17,18,19,20. However, studies on the ultrastructure of hESCs are still very limited.

**Figure 5:**
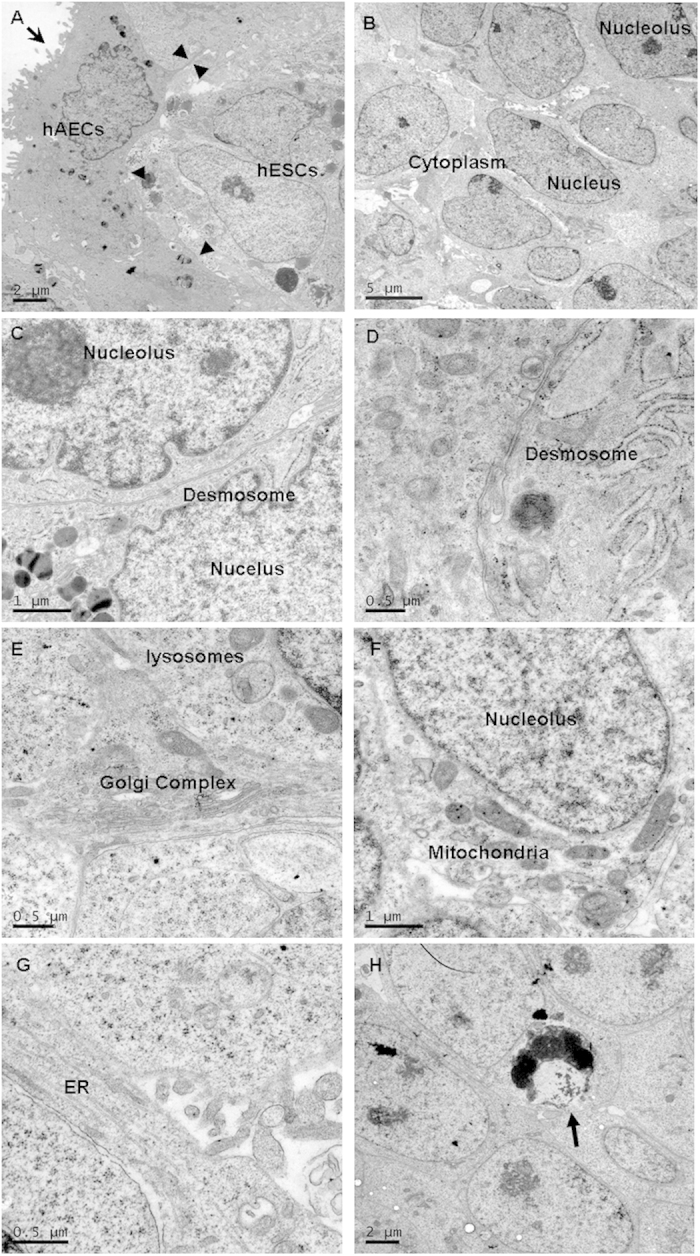
Transmission electron micrographs of GFY-1 on hAECs. (**A**) hESCs on hAECs. The surface of the hAECs contained non-intestinal-type microvilli (arrow). The hAECs exhibited a protein-synthesizing phenotype, based on their large cytoplasm. Cell pseudopodia (arrow head) were observed between hAECs and hESCs. (**B**) hESC colony. In general, hESCs have large nuclei with reticulated nucleoli and scanty cytoplasm. Most of our hESCs exhibited scanty cytoplasm with large nuclei. (**C**,**D**) Desmosomes (marked as “D” in images) are specialized cell contacts and dense cell junctions that were present in our hESC colonies. (**E**) A group of mitochondria was evident in this hESC colony. (**F**,**G**) These cells were generally similar to phagocytic cells with lysosomes, Golgi apparatus, and rough endoplasmic reticulum (ER). (**H**) An apoptotic cell was observed in a hESC colony when the number of passages reached 35. Bars: A, H = 2 μm; B = 5 μm; C = 1μm; D, E, G = 0.5 μm.

TEM showed the surfaces of hAECs to contain non-intestinal-type microvilli, which is consistent with previous report 21. These feeder cells contacted with hESCs through cell pseudopodia. However, no junctions between hAECs and hESCs were observed (Fig. 5A). Consistent with previous studies 17,18,19, the hESCs had large nuclei and scanty cytoplasm, and there were tight junctions and gap junctions between cells (Fig. 5A–D). hAECs had more cytoplasm than hESCs, indicating they were protein-synthesizing cells. The hESCs were close to each other, leaving less extracellular space between adjacent cells (Fig. 5A–B).

The Golgi apparatus and rough endoplasmic reticulum were both clear and well developed, showing typical morphology. Mitochondria, which produced energy in the form of adenosine triphosphate (ATP), were typically organized at the periphery of the nucleus (Fig. 5E–G). It has been suggested that hESCs use this energy for cell survival and self-renewal.

Sathananthan *et al.* reported that differentiated cells are detectable in hESC colonies via TEM. These include fibroblast cells, epithelial cells, and beating cardiac cells 17,20. However, few differentiated cells were found among the hESCs that had been grown on hAECs.

Programmed cell death is common occurrence in ESCs colonies after large numbers of passages 17,20. Apoptotic cells were observed among the hESC colonies after passage 35 (Fig. 5H), but less apoptosis was observed in hESCs of earlier passages.

These results showed the ultrastructures of the hESCs grown on hAECs to be consistent with those of previous reports on undifferentiated. ESCs.

### Pluripotency marker genes on newly derived hESCs grown on hAECs

To further elucidate the pluripotent state of the newly-derived hESC lines, GFY-1 cells maintained on hAECs or mitomycin-C-treated MEFs were grown through 26 passages. HUES-1 cells, which had been donated by the Melton Laboratory of Harvard University (HHMI), were also put through the same number of passages grown on hAECs or MEFs (Fig. S2).

**Figure 6:**
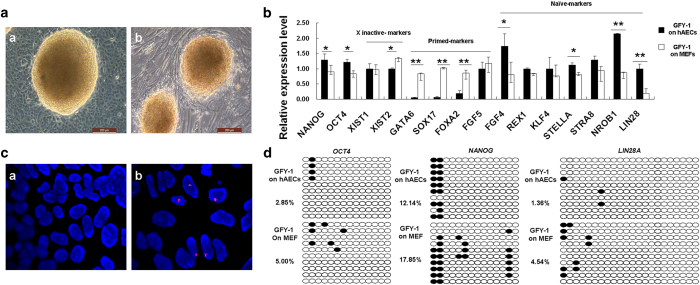
A different pluripotent state was observed in hESCs cultured on hAECs versus MEFs. (**A**) GYF-1 cells of passage 26 on hAECs (a) or on MEFs (b). Bar = 200 mm. (**B**) Expression of pluripotency marker genes was analyzed by real-time PCR. Core pluripotency (*OCT4* and *NANOG*), X inactive, naïve, and primed genes was quantified, 18s rRNA used as the internal control. (**C**) Representative fluorescence in situ hybridization (FISH) analysis for XIST RNA (red) and nuclear DNA (blue). GFY-1 cell line of passage 10 grown on hAECs (a) or on MEFs (b). Original magnification × 1000. (**D**) Methylation analysis of CpG islands within the promoter regions of *OCT4*, *NANOG* and *LIN28A* was done by bisulfite sequencing PCR. Closed black circles indicate methylated loci, and numbers represent ratios of methylated islands.

The GFY-1 cells on hAECs formed dome-like, compact colonies (Fig. 6Aa), but the GFY-1 colonies on MEFs were relatively thin and flattened (Fig. 6Ab). In addition, pluripotency marker genes, including the core pluripotency genes (*OCT4* and *NANOG*), *XIST*-mediated (X inactive-specific transcript) X chromosome silencing genes, primed genes (*GATA6*, *SOX17*, *FOXA2*, and *FGF5*), and naïve genes (*FGF4*, *REX1*, *KLF4*, *STELLA*, *STRA8*, *NR0B1*, and *LIN28*) were analyzed using real-time PCR. Results showed the expression of naïve genes and of *OCT4* and *NANOG* to be significantly more pronounced in GFY-1 cells grown on hAECs than on MEFs. The expression of the primed marker genes, including XIST was significantly lower in GFY-1 cells on hAECs than on MEFs (Fig. 6B, *P* < 0.05 or *P* < 0.01). Further, we tested whether the GFY-1 cells or HUES-1 cells grown on hAECs would reactivate the inactive X chromosome. Results show that hESCs grown on MEFs expressed an XIST cloud in 20%-50% of cells, which is indicative of X inactivation. However, less XIST clouds were seen in hESCs cells on hAECs (Fig. 6C & Fig. S3C), consistent with the observed changes in the mRNA transcript of the XIST (Fig. 6B & Fig. S3A).

Recently, a combination of OCT4, SOX2, NANOG, and LIN28 permitted the reprogramming of human somatic cells to pluripotent stem cells (induced pluripotent stem cells, iPS cells) that exhibit the essential characteristics of ESCs 22. The results of the present work show that mRNA expression of *NANOG*, *OCT4*, and *LIN28* increased in GFY-1 cells on hAECs. For this reason, DNA methylation on promoter regions of these three genes has been subjected to further examination through bisulfite sequencing PCR. Results showed 2.85% of the CpG islands of *OCT4*, 12.14% of the CpG islands of *NANOG*, and 1.36% of the CpG islands of *LIN28* to be partially methylated in GFY-1 cells on hAECs. However, 5% of the CpG islands of *OCT4*, 17.85% of the CpG islands of *NANOG*, and 4.54% of the CpG islands of *LIN28* were found to have been methylated in GFY-1 cells on MEFs (Fig. 6C). Similar results were also observed in HUES-1 cells maintained on hAECs versus MEFs (Fig. S3C). These results showed the methylation of promoters of *NANOG*, *OCT4*, and *LIN28* varied in hESCs in different culture systems.

These findings confirm that the newly derived hESC lines have distinct pluripotent characteristics in different culture system.

## Discussion

Hundreds of hESC lines have been established since the first successful attempt was reported by Thomson *et al.* 1,23. About 210 different derivations are now listed in the NIH registry for research use ( http://escr.nih.gov). Although mESCs and hESCs share many similarities with respect to pluripotency, recent studies have suggested that differences exist between mouse and hESC lines. Ginis *et al.* compared the gene expression profiles of mouse and hESCs, including genetic markers of differentiated and undifferentiated cells; cell proliferation and cell death genes, cell cycle genes, cytokine expression, and the LIFR/gp130 signaling pathway. They showed that the profound differences observed between human and mouse ESCs to be species-specific 8.

As mentioned above, both mESCs and hESCs are derived from the ICM of developing blastocysts. Recently, another type of pluripotent cell has been derived from the postimplantation epiblast of murine embryos. These cells are called epiblast stem cells (mEpiSCs). Studies have demonstrated that mESCs differ from mEpiSCs on both the molecular and epigenetic levels. Mouse EpiSCs and ESCs are considered as a primed and a naïve pluripotent state, respectively 1,24,25. However, hESCs are similar to primed mEpiSCs in that both types of cells appear flattened and are extremely sensitive to a single cell passaging 26, TGFβ/activin-dependent signaling 27, inactivated X chromosomes 28, and BMP4-induced primordial germ cells differentiation *in vitro* 29. This suggests that the underlying reasons for the different characteristics of different ESCs cannot be explained by species differences alone. Some studies have suggested that culture conditions for growing hESCs were not optimized for the maintenance of cells in an undifferentiated state and that the differences observed here might be resolved as culture conditions improve 8,10,11. Hanna *et al.* were the first to report the reprogramming of hESCs to the ground naïve pluripotent state using ectopic induction factors. In these ways, culture conditions have been found to influence the type of pluripotency displayed by ESCs 4. In this way, optimization of growth conditions is relevant to any application in which enhanced, long-term, or stable pluripotency is necessary.

Our previous work demonstrated that using hAECs as a feeder layer is an ideal strategy for propagating hESCs. This strategy not only limits contact with animal products, but also better maintains the pluripotency of ESCs. The hESCs grown on hAECs formed packed colonies, unlike the flat colonies formed on MEFs. Besides this, epigenetic changes of gene expression in hESCs on hAECs show that hESCs have a distinct characteristic 9,10,11,12. Consistent with this, new hESC lines derived on hAECs culture system demonstrated their ability to undergo long-term undifferentiated growth and development *in vitro* and *in vivo*. More importantly, domed-shaped colonies of hESCs are usually observed in hAECs culture system. Recently, Gafni *et al.* established and derived novel human ground state naïve pluripotent stem cells. They clearly demonstrated that these human naïve pluripotent stem cells have domed-shaped colonies resembling murine naive ESCs and distinct from conventional primed human pluripotent cells which usually maintained on common culture system 30. Evidence was further demonstrated recently by Theunissen *et al.* 31.

Although the ultrastructures of mESCs and hESCs have been documented, the present study is the first to demonstrate ultrastructural features of hESCs on hAECs. Here, results showed that the hESC colonies consisted of mostly undifferentiated cells and far fewer apoptotic cells as the number of passages increased than in a comparable system reported by Sathananthan *et al.* 17,20. Programmed cell death is common in ESC colonies. It was demonstrated that the apoptosis of ESCs could be induced when the cells were dispersed as single cells. However, apoptosis was blocked when ESCs were cultured on feeders. Soluble factors secreted from feeder cells did not block the apoptosis of ESCs suggesting that direct interaction between ESCs and feeders is required for the blockage of apoptosis 32. Specifically, hESC colonies were found to include undifferentiated, differentiating, and differentiated cells. A significant increase in the rate of spontaneous differentiation was observed in the periphery of the colonies during the later stages of culture growth 19,20. However, unlike hESCs, mESC colonies stably contain mostly undifferentiated cells 18. In this way, it seems that hAECs is suitable to maintain the undifferentiated growth of hESCs.

The relationship between hAECs and hESCs was evaluated. The feeder cells were found to contact with hESCs through cell pseudopodia. Desmosomes, which are the common junctions between hESCs, were not observed between hAECs and hESCs. During hESC culturing, hESC clones were found to be easily separated from hAECs, and less contamination was observed in the hAECs. However, hESC maintained on MEFs were always mixed with MEFs 9,10. Thus, RT-PCR was done to test the contamination of hESCs with hAECs (Fig. S4). Epithelial marker E-caderin and neural-specific gene expression markers, such as neurone-specific enolase (NSE) and myelin basic protein (MBP) which specifically expressed in hAECs 13 was assayed in newly derived hESCs, however, these genes were not detected in undifferentiated hESCs separated from feeder layers. This further demonstrated the superiority of hAECs as a feeder layer.

The hESCs in the present study showed large nuclei containing reticulated nucleoli, well-developed rough endoplasmic reticula (RER), Golgi complexes, elongated tubular mitochondria, lysosomes, and cytoskeletons. These observations were largely consistent with those of the other groups 17,18,19,20. Very large nuclei and scanty cytoplasm with fewer organelles are characteristics of undifferentiated ESCs 17,18,19,20. In this way, analyses of morphological features are necessary reasonable understanding of the undifferentiated growth state of hESC colonies.

While many genes are important for establishing and maintaining the pluripotency of ESCs, several transcriptional regulators are specifically associated with the pluripotent state. These factors include the homeodomain-containing proteins Oct4 and Nanog 26,27. Recent studies have shown that the RNA-binding protein Lin28 can take part in production of human iPS cells from somatic cells 22. Lin28 can also prevent human and mouse stem cells from differentiating 33,34. Here, the expression of pluripotency marker genes by the newly derived hESC colony grown on hAECs was compared to expression of these marker genes by GFY-1 cells grown on MEFs. Results showed that both GFY-1 cells and HUES-1 cells grown on hAECs exhibited higher expression of naïve marker genes than those grown on MEFs, but the expression of primed genes was dramatically lower in hESCs grown on hAECs. The core pluripotent markers, including OCT4, NANOG, and LIN28, showed higher expression levels in hESCs grown on hAECs than those on MEFs.

Targeted demethylation of Oct4 and Nanog has been shown to take place in ESCs. In this way, the ESCs become hypermethylated during differentiation. This suggests that this process affects the network regulators that maintain pluripotency 35. Lin28 and similar RNA-binding proteins may be suitable for use as markers pluripotency because of the role that they play in the regulation of cell growth 22. In this way, RNA-induced transcriptional silencing of LIN28 may also be responsible for inactivating its promoter through an altered epigenetic state. Hypermethylation of the LIN28 promoter sequence and repression of its transcript have been detected in differentiated embryonic carcinoma cells and mesenchymal stem cells 36. Here, reduced methylation of these core genes accompanied by the upregulation of *OCT4*, *NANOG*, and *LIN28* were observed in hESCs grown on hAECs. It is here suggested that methylation status differs in hESCs derived and propagated on different feeder layers.

The improvements in the optimized culture conditions for naive hESCs may provide a better platform for assessing the developmental potential of naive hESCs in vitro and in vivo 30,31. In current regular culture system, most of femal hESCs have already undergone X inactivation, whereas X-chromosome inactivation represents an important epigenetic difference between the naïve and primed states of pluripotency 4. Herein, we indicated that both X chromosomes of the female hESCs derived and cultured on hAECs would be activated, further demonstrating that hAECs feeder layers could maintain naïve-like property of hESCs. Gafni *et al.* found that TGF-β1 cytokine supplementation were essential to maintain naive human pluripotent stem cells 30, and we also provide the evidence that concentration of TGF-β1 cytokine secreted by hAECs feeder layer is much higher than that of MEFs feeder layer (Fig. S5). Although more rigorous evidences are still needed to demonstrate the characteristics of ground state naïve pluripotent stem cells, compared with the complex genetic manipulations as well as the screening of chemicals and growth factors, the hAECs culture system is more convenient to maintain the naïve-like state of hESCs.

So far, the cultures of human pluripotent stem cells maintained on MEF are still widely used. To avoid the risks of zoonosis, it is important to derivate of human cell-based feeder to support the propagation of human ESCs. Many types of human cells have been used as feeder layers, such as human fibroblasts, fetal muscles, fetal skin, adult fallopian tube epithelial, adult uterine endometrium, adult marrow stromal cells, but most of these are difficult to acquire due to ethical and practical limitations 37,38,39,40,41. Although feeder-free cultures of hESCs are reported, the chromosomal instabilities of hESCs happened during the long time culture 42. In contrast, the human placental amnion is abundantly available as routinely discarded tissues, and there are no ethical complications. hAECs, unlike the MEF, do not need to be pretreated by mitomycin C or gamma irradiation due to the slow cell growth and are ready to be as feeder layer 9,10,11,12. Thus, from the perspective of source, the hAECs could be better candidates for generating feeder layers for hESC derivation and culture.

In summary, this paper describes the successful establishment of four hESC lines using hAECs as a feeder layer. The ultrastructure of these hESCs indicated that an undifferentiated growth state could be maintained on hAECs. We further showed that hESCs grown in a hAEC-based culture system exhibited distinctive characteristics. This type of optimized culture system may provide a useful platform for establishing clinical-grade hESCs and assessing the undifferentiated potential of hESCs.

## Methods

### Preparation of hAECs

Human placentas were collected from healthy mothers who provided written informed consent, after uncomplicated elective Cesarean section. The procedure was approved by the ethics committee of the international peace maternity and child health hospital, school of medicine, Shanghai Jiaotong University. All donors were negative for hepatitis A, B, C, and D, HIV-I, and TPAB (Treponema pallidum antibody) with approval from the institutional ethics committee and written informed consent were obtained. Cultures of hAECs were prepared as previously described 9. Briefly, human amniotic membranes were separated from the placental chorions. They were then washed with phosphate-buffered saline. Cells were dissociated from the human amnion using trypsin; cultured in DMEM/F-12 medium (Gibco, Grand Island, NY, USA ) supplemented with 10% knockout (KO) serum replacement (Gibco, Grand Island, NY, USA), streptomycin (100 U/mL), penicillin (100 U/mL), and glutamine (0.3 mg/mL); and incubated in a humidified tissue culture incubator containing 5% CO_2_ at 37 °C. The hAECs were grown to 70–80% confluence and then used as feeder layers for hESC culture. Methods were carried out in accordance with the approved guidelines.

### Derivation of hESCs

Frozen human embryos have been produced by *in vitro* fertilization (IVF) for clinical purposes and donated by individuals (average age 30.5 ± 4.5 years) who provided written informed consents. The procedure was approved by the ethics committee of the international peace maternity and child health hospital, school of medicine, Shanghai Jiaotong University and written informed consent were obtained. Eight donated embryos were frozen on Day 2 and cultured as described previously 1. Blastocysts with intact zona were treated in protase for 1–3 min (10 U/mL; Sigma-Aldrich, USA). The ICMs were then isolated from the blastocysts and plated onto the hAEC feeder layers. Cells were cultured in DMEM/F-12 medium containing 20% KO serum replacement (Life Technologies, Carlsbad, CA, USA), 2 mM L-glutamine (Life Technologies, Carlsbad, CA, USA), 1% non-essential amino acids (Life Technologies, Carlsbad, CA, USA), 0.1 mM β-mercaptoethanol, and 10 ng/mL human recombinant bFGF (Life Technologies, Carlsbad, CA, USA). After the appearance of outgrowth cells (approximately 1–2 weeks), cells were mechanically dissected and transferred onto fresh layer cells. The culture medium was changed every day. The hESCs were passaged every 7–8 days. Methods were carried out in accordance with the approved guidelines.

### Immunofluorescent staining

The Human Embryonic Stem Cell Marker Antibody Panel (R&D systems, MN, USA) was used to characterize hESCs. Immunofluorescent staining was performed according to the instructions. FITC-conjugated secondary antibodies (Santa Cruz, CA, USA) were used to label the primary antibodies. Nuclei were stained with DAPI (4′, 6-diamidino-2-phenylindole, Santa Cruz, CA, USA).

Goat polyclonal antibodies against nestin (1:200, Santa Cruz, CA, USA), goat polyclonal antibodies against Sox-17 (1:200, Santa Cruz, CA, USA), and mouse monoclonal antibodies against brachyury (1:200, Santa Cruz, CA, USA) were used to mark differentiated cells. These markers were then visualized with FITC-conjugated secondary antibodies (Santa Cruz, CA, USA). Nuclei were stained with DAPI (Santa Cruz, CA, USA).

### *In vitro* differentiation assay

The *in vitro* differentiation assay was performed as described previously 12. Cell clones were picked and allowed to develop into embryoid bodies in differentiation medium. Embryoid bodies were then transferred to gelatin-coated plates for further differentiation, then were ultimately harvested for RNA extraction or fixed with 4% paraformaldehyde for immunostaining.

### Teratoma formation

All animal procedures were conducted according to experimental protocols previously approved by the Institutional Animal Care and Use Committee of Shanghai Jiaotong University. After 20 passages on hAECs, 2 × 10^6^ hESCs were injected into the hind legs of severe combined immunodeficient (SCID) mice. After 8–10 weeks, tumors were embedded in paraffin and analyzed via hematoxylin and eosin (H&E) staining.

### RT-PCR

Total RNA was extracted from fresh cells with Trizol (Life Technologies, Carlsbad, CA, USA) then reverse-transcribed into cDNA with a ReverTra Aca-α Kit (Toyobo, Japan). RT-PCR was performed according to standard procedures and the products were electrophoresed on a 2% agarose gel. Primer pairs used in this study are list in Supplemental Table 1.

### Karyotype analysis

For chromosome analysis, hESCs in the exponential growth stage were treated for 2 h with colcemid (0.1 mg/mL). After colcemid treatment, digested single cells were karyotyped using the G-band method.

### Transmission electron microscopy (TEM)

For ultrastructure analysis, hESCs were grown on hAEC feeder layers on glass coverslips. They were fixed with 2.5% glutaraldehyde in phosphate-buffered saline (0.1 M, pH 7.4) for 24 h at 4 °C, and then postfixed with 1% osmium tetroxide in the same buffer for 1 h at room temperature. Samples were dehydrated and embedded in epoxy resin. Ultrathin sections were counterstained with lead citrate and uranyl acetate. Ultrastructure visualization was performed using an energy-filtered transmission electron microscope (Tecnai G2 Spirit TEM, FEI Company, USA).

### Quantitative real-time PCR and DNA methylation analysis

For qPCR, each sample was analyzed in triplicate, with 18S rRNA as the internal control, using IQ SYBR Green (Bio-Rad, CA, USA). Amplification data were collected and analyzed using a Mastercycler ep realplex Realtime PCR system (Eppendorf, Hamburg, Germany).

Genomic DNA from hESCs on hAECs or on MEFs was treated with sodium bisulfite, according to protocol. Treated DNA was subjected to RT-PCR. The PCR products were cloned into T-vectors (Promega, WI, USA) and individually sequenced with primers for the promoter regions of *OCT4 (POU5F1)*, *NANOG*, and *LIN28A*. Primer information is provided in Supplemental Table 2.

### XIST RNA FISH hybridization

Human embryonic stem cell (hESCs) on hAECs or on MEFs were grown on chamber slides (BD Falcon, USA) for 24 h and then fixed in 4% formaldehyde for 15 min. The cells were permeabilized in PBS containing 0.5% Triton-X for 5 min on ice and washed in PBS and 2 × SSC. The the localization of XIST RNA was detected using a direct-labeled RNA FISH probe purchased from Cambio (Cambridge, UK) and was hybridized according to the manufacturer’s instructions. The nuclei were counterstained with DAPI and viewed with BX21 fluorescent microscope (Olympus, Japan). Images were captured with a FISH progress (imstar, France).

### Statistical analysis

Experimental data are expressed as mean ± SD. The statistical significance of the differences was assessed with the Student’s t-test. Differences were considered statistically significant when *P* < 0.05.

## Author Contributions

D.L. designed the study, Y.C. and T.L., participated in the data collection, statistical analysis, and drafted the manuscript. D.L., Y.W., J.S. and Y.W. participated in data collection, interpretation of data and statistical analysis. D.L., L.G. and C.W. conceived the study, participated in its design and coordination, and revised the manuscript. All the authors read and approved the final version of the manuscript.

## Additional Information

**How to cite this article**: Lai, D. *et al.* Derivation and characterization of human embryonic stem cells on human amnion epithelial cells. *Sci. Rep.* **5**, 10014; doi: 10.1038/srep10014 (2015).

## Supplementary Material

Supporting Information
