# Supplementary material for: Derivation and characterization of human embryonic stem cells on human amnion epithelial cells
Source: Sci Rep. 2015 May 7;5:10014. doi: 10.1038/srep10014 (PMC4423442; doi:10.1038/srep10014)
Supplement: Supporting Information [file srep10014-s1.pdf]

Supplementary information

Derivation and characterization of human embryonic stem cells on human amnion epithelial cells

Dongmei Lai #1, Yongwei Wang #1, Jian Sun #1, Yifei Chen 1, Ting Li 1,Yi Wu 1, Lihe Guo 1, Chunsheng Wei 2

1. The International Peace Maternity and Child Health Hospital, School of medicine, Shanghai Jiaotong University, Shanghai, China.
2. Eye and ENT Hospital, Fudan University, Shanghai, China

# Contributed equally
Correspondence:
To whom correspondence should be addressed to:
Dr. Dongmei Lai or Dr. Chunsheng Wei

Methods

ELISA assay

Cell culture supernatant was collected from hAECs and MEFs respectively. TGF-beta1 secreted in these supernatant was measured using a TGF-beta1 ELISA kit (R&D systems, Minneapolis, MN, USA) according to the manufacturer's instructions. The experiment was independently repeated three times.

Figure and Figure legends

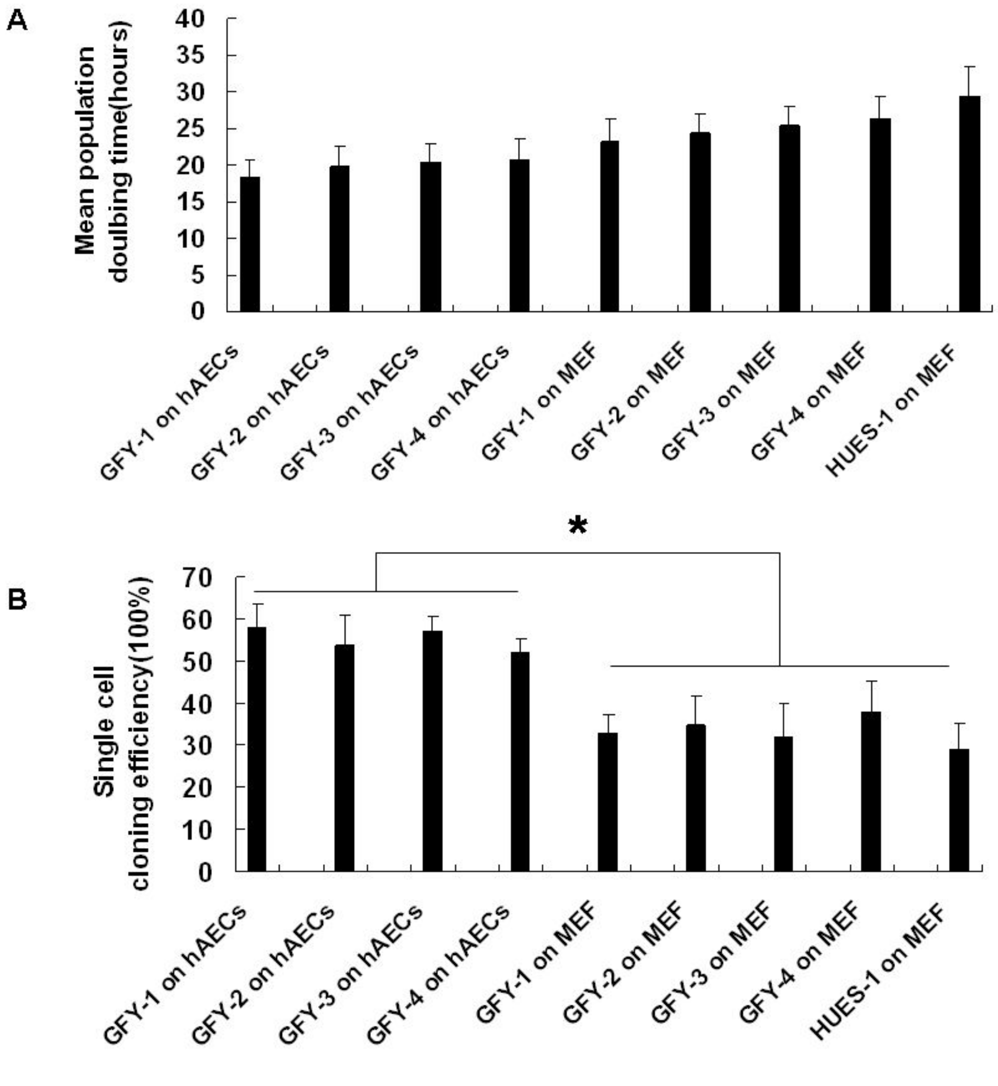

Supplemental Figure 1. Growth properties of hESCs in different feeder cells. A, Population doubling time of the newly derived hESCs and human ESC line HUES-1 maintained on hAECs or MEF. After plating each cell line in replicates, cells were collected at days 2, 4 and 6 and their growth rates were normalized by the number of cells counted at day 2. Error bars represent SD (n=3). B, Single-cell cloning efficiency of human ESC lines on different feeders was determined by the number of wells containing colonies 7 days after plating. (Error bars indicate SD, n=3, * $P < 0.05$)

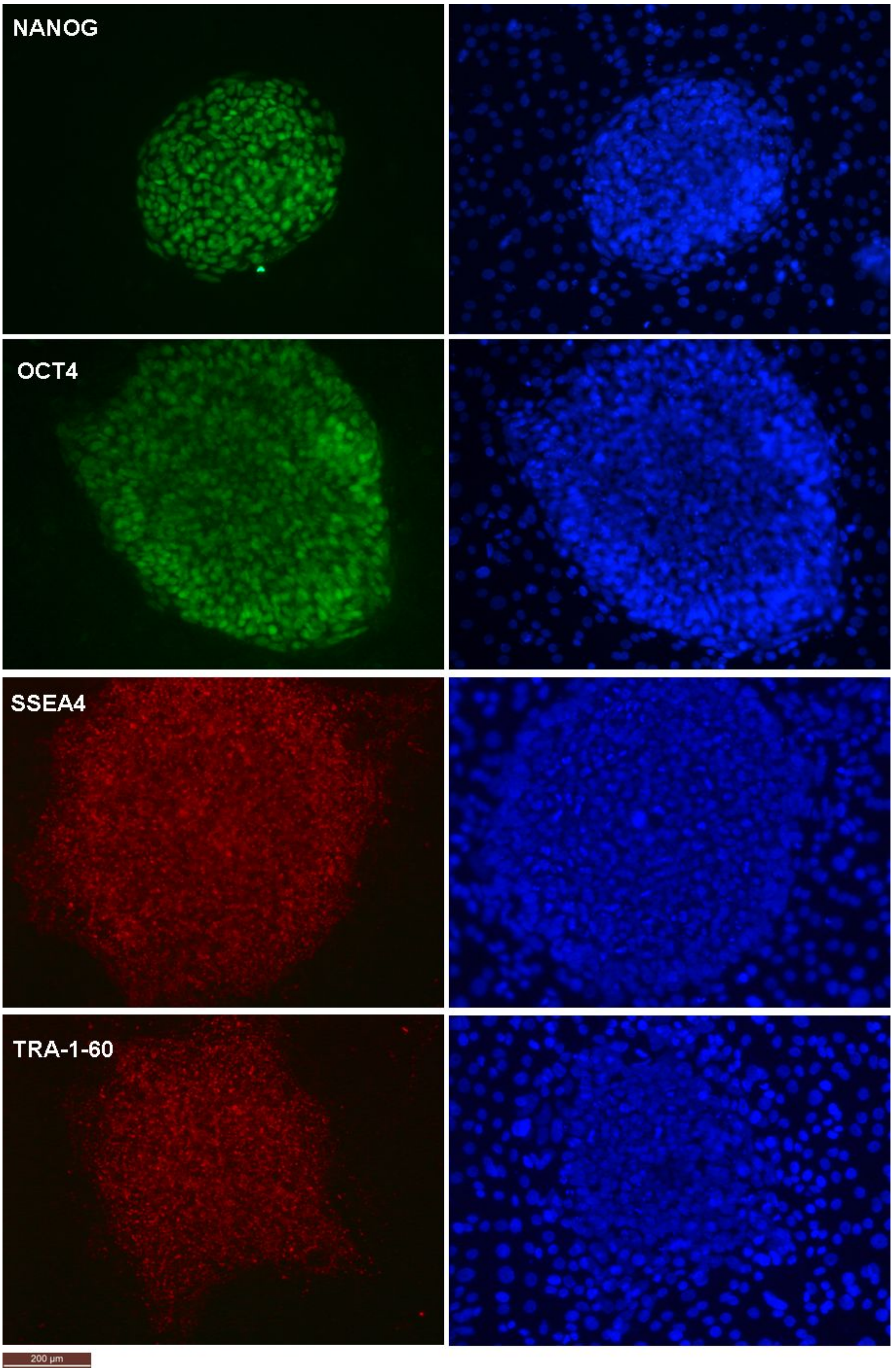

Supplemental Figure 2. The expression of undifferentiated markers was detected in HUES-1 cells maintained on MEF.

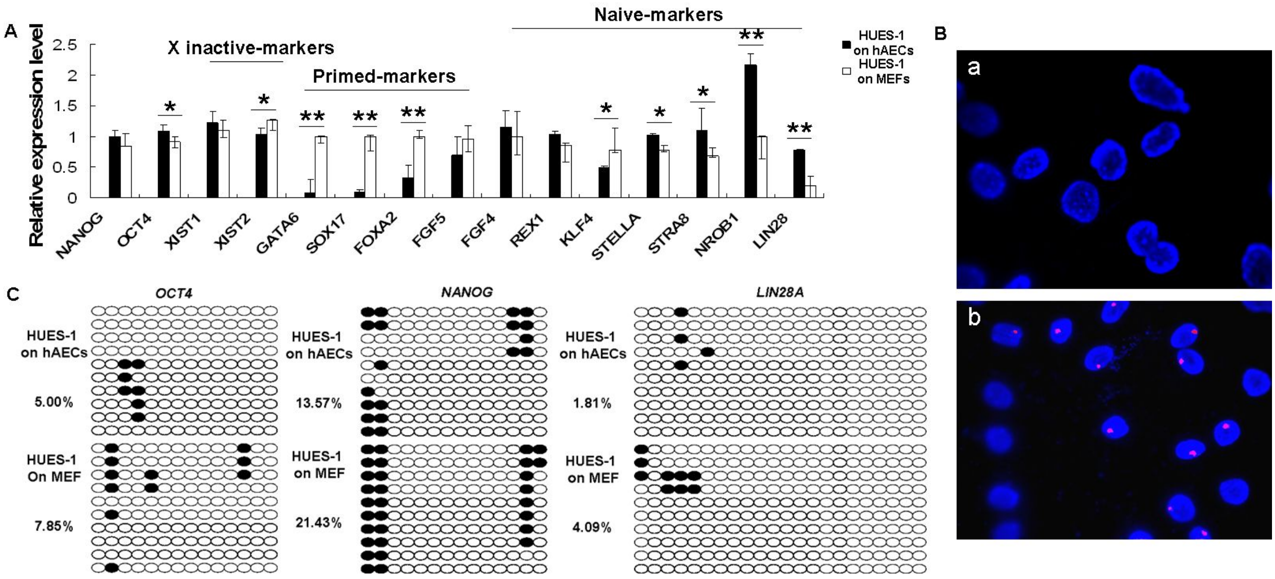

Supplemental Figure 3. A different pluripotent state was also observed in HUES-1 maintained on hAECs versus MEF. A) Expression of pluripotent marker genes, including core pluripotency (*OCT4* and *NANOG*), X inactive, naïve, and primed genes, was quantified by real-time PCR, 18s rRNA used as the internal control. B) Representative FISH analysis for XIST RNA (red) and nuclear DNA (blue). HUES-1 cell line of passage 6 grown on hAECs (a) or on MEFs (b). Original magnification ×1000. C) Methylation analysis of CpG islands within the promoter regions of *OCT4*, *NANOG* and *LIN28A*. Closed black circles indicate methylated loci, and numbers represent ratios of methylated islands.

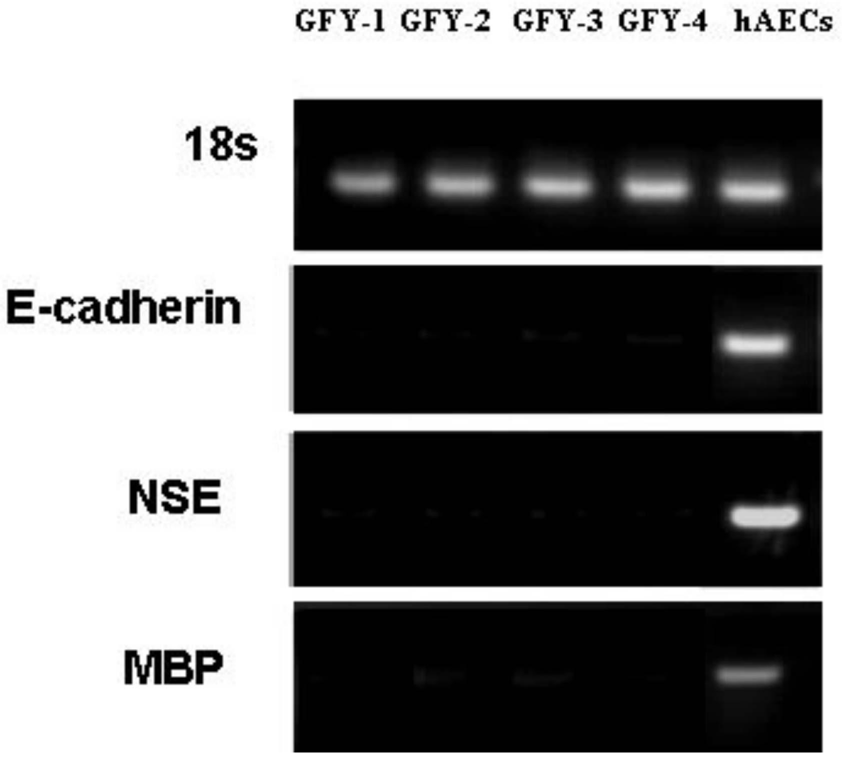

Supplemental Figure 4. Less contamination was observed in the newly derived human ES cells on hAECs. Epithelial marker E-caderin and neural-specific gene expression markers, such as neurone-specific enolase (NSE) and myelin basic protein (MBP), was detected in hAECs by RT-PCR, however, these genes were not detected in undifferentiated human ES cells separated from feeder layers.

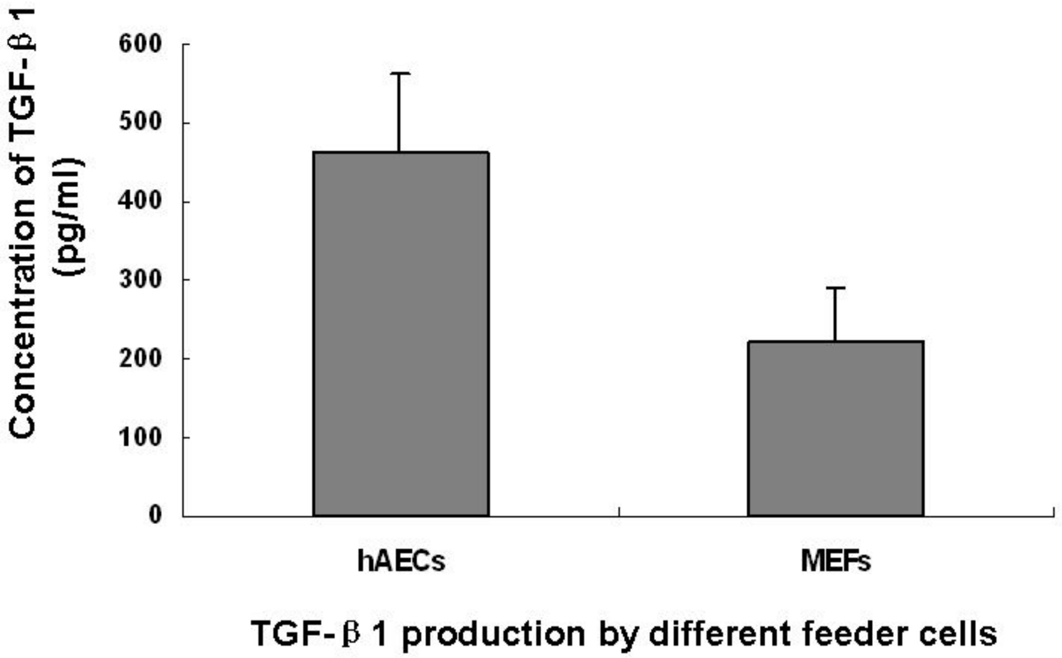

Supplemental Figure 5. TGF-β1 production by different feeder cells was assayed by ELISA. The concentration of TGF-β1 secreted by hAECs was significantly higher than that in MEFs (*P<0.01*).

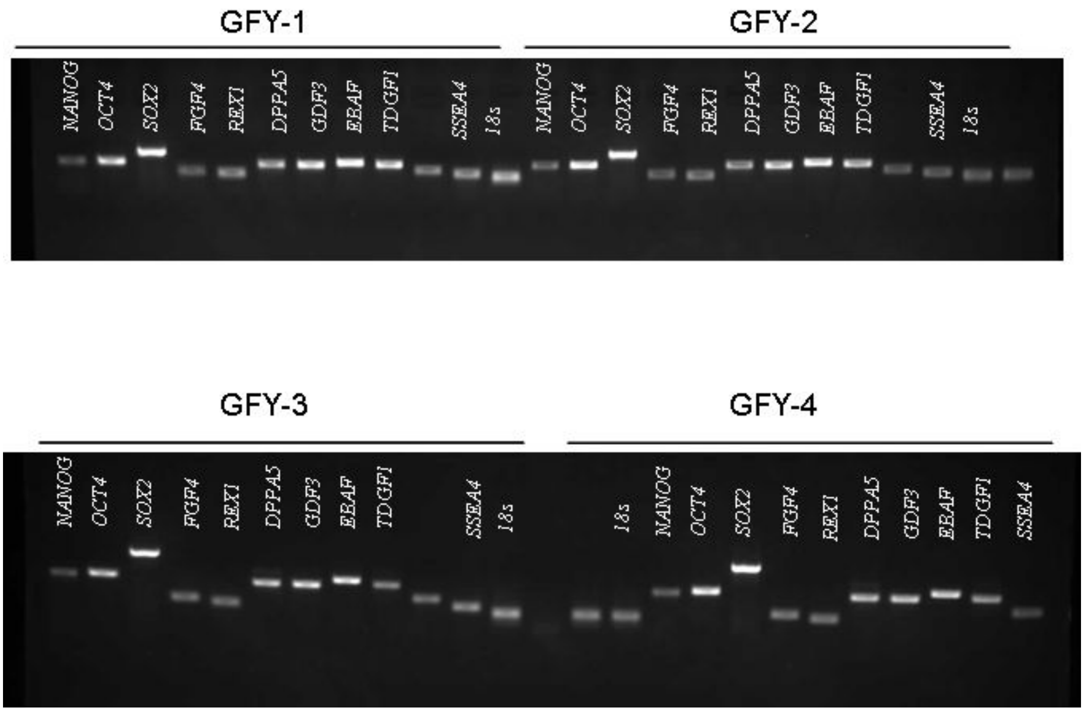

Supplemental Figure 6. Original PCR scans for Figure 2

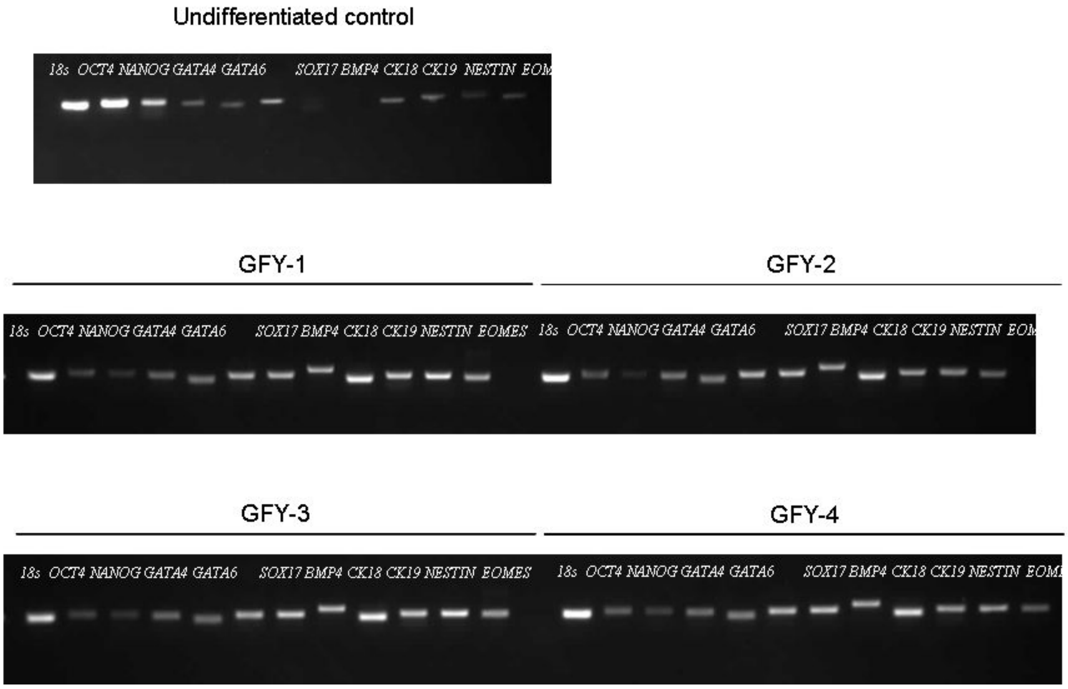

Supplemental Figure 7. Original PCR scans for Figure 4

Tables

Supplemental Table 1. Primers for RT-PCR.

Gene name	forward primer (5′→ 3′)	reverse primer (5′→ 3′)
NANOG	TTCCTTCCTCCATGGATCTG	TCTGCTGGAGGCTGAGGTAT
OCT4	ATCCTGGGGGTTCTATTTGG	CTCCAGGTTGCCTCTCACTC
SOX2	ACACCAATCCCATCCACACT	GCAAACTTCCTGCAAAGCTC
FGF4	GATGAGTGCACGTTCAAGGA	GGTTCCCCTTCTTGGTCTTC
REX1	ATGGCGTCCAAGACTACCAC	ACTTTGCCCCCAAACTCTTT
DPPA5	CCGAAGACCTGAAAGATCCA	GACTGGAGCATCCACTTGGT
GDF3	GCCATCAAAGAAAGGGAACA	GCAGGTTGAAGTGAACAGCA
EBAF	GCTGAGCAATGCACACATTT	GAAGCCCTTCATCCTTCCTC
TDGF1	ACAGAACCTGCTGCCTGAAT	ATCACAGCCGGGTAGAAATG
SSEA4	TGGACGGGCACAACTTCATC	GGGCAGGTTCTTGGCACTCT
18s rRNA	CGTTGATTAAGTCCCTGCCCTT	TCAAGTTCGACCGTCTTCTCAG
GAPDH	AGGTCGGTGTGAACGGATTTG	GGGGTCGTTGATGGCAACA
GATA6	TCCACTCGTGTCTGCTTTTG	CCCTTCCCTTCCATCTTCTC
SOX17	CCTGGGTTTTTGTTGTTGCT	GAGGAAGCTGTTTTGGGACA
T	ACCCAGTTCATAGCGGTGAC	ATGAGGATTTGCAGGTGGAC
BMP4	TTTGTTCAAGATTGGCTGTC	AGATCCCGCATGTAGTCC
CK18	CACAGTCTGCTGAGGTTGGA	GAGCTGCTCCATCTGTAGGG
NESTI	AACAGCGACGGAGGTCTCTA	TTCTCTTGTCCCGCAGACTT
EOMES	CCACTGCCCACTACAATGTG	TTCCCGAATGAAATCTCCTG
E-caderin	GCCTCCTGAAAAGAGAGTGGAAG	TGGCAGTGTCTCTCCAAATCCG
NSE	CCCACTGATCCTTCCCGATACAT	CCGATCTGGTTGACCTTGAGCA
MBP	TTAGCTGAATTCGCGTGTGG	GAGGAAGTGAATGAGCCGGTTA

Supplemental Table 2. Primers for qRT - PCR and genomic PCR

Gene name	forward primer (5′→ 3′)	reverse primer (5′→ 3′)
NANOG	GGGCCTGAAGAAAACTATCCATCC	TGCTATTCTTCGGCAGTTGTTTT
OCT4	GGCCCGAAAGAGAAAGCGAACC	ACCCAGCAGCCTCAAAATCCTCTC
XIST1(Exon1-2)	GAAGAGTCTCTGGCTCTTTAGAATACTGA	CAGCGTGGTATCTTCAATGGG
XIST2 (Exon5-6)	GCCTGGCACTCTAGCACTTGA	AAGAGACAAAGAAATACACATTCATTCAG
GATA6	TCCACTCGTGTCTGCTTTTG	CCCTTCCCTTCCATCTTCTC
SOX17	CCTGGGTTTTTGTTGTTGCT	GAGGAAGCTGTTTTGGGACA
FOXA2	CTACGCCAACATGAACTCCA	CGGTAGAAGGGGAAGAGGTC
FGF5	CTTGGAGCAGAGCAGTTTCC	CTTCGTGGGATCCATTGACT
FGF4	GATGAGTGCACGTTCAAGGA	GGTTCCCCTTCTTGGTCTTC
REX1	ATGGCGTCCAAGACTACCAC	ACTTTGCCCCCAAACTCTTT
KLF4	CCCACACAGGTGAGAAACCT	ATGTGTAAGGCGAGGTGGTC
STELLA	CTCAAATCTCCTCCGAGACG	TTCGATTTCCCTGAGGACTG
STRA8	TCGTCTCCGCGGCCATCTCC	TGTCCTTCACGCTGCCCTCG
NR0B1	GTGAACACACCAGGATGACG	ATGATGGGCCTGAAGAACAG
LIN28	AGTGGCCTGGATAGGGAAGT	CTTGGCTCCATGAATCTGGT
18s rRNA	CGTTGATTAAGTCCCTGCCCTT	TCAAGTTCGACCGTCTTCTCAG
OCT4 (Bisulfate seq)	GATTTGTATTGAGGTTTTGGAGG	AAACCTTAAAAACTTAACCAAATCC
NANOG (Bisulfate seq)	TGTTGTTTAGGTTGGAGTATAGTG	CCCTTATAAATTCTCAATTAATCC
LIN28A (Bisulfate seq)	GGGATATTTTAGAGGTGTTAGAGAT	TCCCTTCTACRTAACTCCTAACC
